# Associations between Physical Activity and Obesity Defined by Waist-To-Height Ratio and Body Mass Index in the Korean Population

**DOI:** 10.1371/journal.pone.0158245

**Published:** 2016-07-22

**Authors:** On Lee, Duck-chul Lee, Sukho Lee, Yeon Soo Kim

**Affiliations:** 1 Department of Physical Education, College of Education, Seoul National University, Seoul, South Korea; 2 Department of Kinesiology, College of Human Sciences, Iowa State University, Ames, Iowa, United States of America; 3 Department of Counseling, Health, and Kinesiology, College of Education and Human Development, Texas A&M University-San Antonio, San Antonio, Texas, United States of America; University of the Balearic Islands, SPAIN

## Abstract

**Objective:**

This study investigated the associations between physical activity and the prevalence of obesity determined by waist-to-height ratio (WHtR) and body mass index (BMI).

**Methods:**

This is the first study to our knowledge on physical activity and obesity using a nationally representative sample of South Korean population from The Korea National Health and Nutrition Examination Survey. We categorized individuals into either non-obese or obese defined by WHtR and BMI. Levels of moderate-to-vigorous physical activity were classified as ‘Inactive’, ‘Active’, and ‘Very active’ groups based on the World Health Organization physical activity guidelines. Multivariable logistic regression was used to examine the associations between physical activity and the prevalence of obesity.

**Results:**

Physical activity was significantly associated with a lower prevalence of obesity using both WHtR and BMI. Compared to inactive men, odds ratios (ORs) (95% confidence intervals [CIs]) for obesity by WHtR ≥0.50 were 0.69 (0.53–0.89) in active men and 0.76 (0.63–0.91) in very active men (p for trend = 0.007). The ORs (95% CIs) for obesity by BMI ≥25 kg/m^2^ were 0.78 (0.59–1.03) in active men and 0.82 (0.67–0.99) in very active men (p for trend = 0.060). The ORs (95% CIs) for obesity by BMI ≥30 kg/m^2^ were 0.40 (0.15–0.98) in active men and 0.90 (0.52–1.56) in very active men (p for trend = 0.978). Compared to inactive women, the ORs (95% CIs) for obesity by WHtR ≥0.50 were 0.94 (0.75–1.18) in active women and 0.84 (0.71–0.998) in very active women (p for trend = 0.046). However, no significant associations were found between physical activity and obesity by BMI in women.

**Conclusions:**

We found more significant associations between physical activity and obesity defined by WHtR than BMI. However, intervention studies are warranted to investigate and compare causal associations between physical activity and different obesity measures in various populations.

## Introduction

Obesity is an escalating problem worldwide as a major risk factor in many serious chronic diseases including cardiovascular disease, dyslipidemia, hypertension, type 2 diabetes, some forms of cancer, and osteoarthritis [1, 2]. The lack of physical activity is one of the main risk factors that lead to overweight and obesity [3, 4]. The average weight gain of normal adults is approximately 1 pound per year, at least over the past decade in American and Australian [5, 6]. The level of physical activity may impact the amount of weight gain occurring over time [4]. However, most studies on physical activity and obesity have been conducted in western populations, and this is the first study on physical activity and obesity in a nationally representative Korean population. Based on the important differences in socioeconomic factors, lifestyle, and culture between Koreans and western populations, this study will add our knowledge in obesity prevention in Asian populations.

Body mass index (BMI), as the individual's body weight in kilograms divided by the square of the height in meters (kg/m^2^), has been widely used to define obesity across populations. However, several studies have shown that waist-to-height ratio (WHtR) is a better predictor of cardiovascular disease than BMI [7–10]. Various cut-off points for WHtR have been suggested, and WHtR can be a good candidate as an indicator of excessive abdominal body fat. BMI depends only on the weight and height of a person, and it does not consider the distribution or composition of body weight.

Also, BMI cut-off value (≥30 kg/m^2^) to diagnose obesity has a high specificity, but low sensitivity to identify adiposity, as they fail to identify half of the people with excess percent body fat [11]. Moreover, visceral rather than subcutaneous fat accumulation mostly around the waist is associated with increased secretion of free fatty acids, hyperinsulinemia, insulin resistance, hypertension, and dyslipidemia [12, 13]. In addition, given the same BMI, Asians have a higher percentage of body fat, increased abdominal obesity, higher levels of intramyocellular lipids, and/or a higher liver fat content compared to Caucasians [14, 15]. These characteristics can contribute to a higher predisposition to insulin resistance and other metabolic diseases at a lesser degree of obesity in Asians compared to Caucasians. Based on these ethnic differences, abdominal obesity defined by WHtR may be a better indicator of obesity in Asian societies.

However, most previous studies on the associations between physical activity and obesity predominantly used BMI to define and diagnose obesity, but did not include WHtR. The purpose of this study was to investigate the associations between physical activity and the prevalence of obesity defined by both WHtR and BMI in the Korean population.

## Materials and Methods

### Participants

We obtained data from the Fourth Korea National Health and Nutrition Examination Survey (KNHANES) in 2007–2009. This survey was conducted as a cross-sectional health survey and used a stratified, multistage, cluster probability sampling method to select a representative sample of the non-institutionalized Korean population by the Korean Center for Disease Control and Prevention. The KNHANES is composed of three examinations: a health interview, health examination, and nutrition survey. The health interview and health examination are performed by trained medical staff and interviewers at the mobile examination center (survey truck) similar to the US NHANES. One week after the health interview and health examination, dieticians visit the houses of participants for the nutrition survey [16]. The total of 24,871 individuals completed the survey with 78.4% response rate. All participants were informed about the purpose and procedure of the study, and provided written informed consent. The study protocol was approved by the Korean Ministry of Health and Welfare and conducted in accordance with the Ethical Principles for Medical Research Involving Human Subjects, as defined by the Helsinki Declaration. After excluding individuals who engaged regular physical activity less than one year, had any history of cardiovascular diseases or cancer, did not complete major survey questions, or were pregnant women, the final sample included 7221 (3350 men and 3871 women) individuals with complete data for the analyses.

### Physical activity assessment and classification

Participants were asked their physical activity levels during a normal week. Regarding moderate physical activity, participants were asked the following question: ‘how many days during the past week did you engage in moderate physical activity (for at least 10 minutes) that caused a slight increase in your breathing or heart rate (e.g. tennis doubles, volleyball, badminton or table tennis, or any other activity)?’ Vigorous physical activity question was, ‘how many days during the past week did you engage in vigorous physical activity such as running, mountain climbing, soccer, basketball or any other activity (for at least 10 minutes) that caused a substantial increase in your breathing or heart rate?’ For each moderate and vigorous activity, the respondents indicated the number of days per week (frequency) and the total time per day (duration) devoted to the activity. To calculation the amount (min/week) of each moderate and vigorous physical activity, the frequency was multiplied by the duration. The total amount of moderate-to-vigorous physical activity was then calculated as the sum of the moderate and vigorous physical activity.

The level of physical activity was classified into three groups based on the World Health Organization (WHO) physical activity guidelines, which is at least 150 minutes of moderate or 75 minutes of vigorous intensity aerobic activity per week, or an equivalent combination of both [17]. Participants who did not meet the guidelines were classified as ‘inactive’ group. Participants who engaged in 150–299 min/week of moderate or 75–149 min/week of vigorous activity were classified as ‘active’ group. Participants who engaged in ≥300 min/week of moderate or ≥150 min/week of vigorous activity were classified as ‘very active’ group. Regarding the total amount of moderate-to-vigorous physical activity, the duration of vigorous activity was multiplied by two based on the guidelines, indicating that one minute of vigorous activity is equal to two minutes of moderate activity.

### Obesity measures

Height and weight were measured with the subjects wearing light clothing and no shoes using SECA 225 height rods (SECA, Hamburg, Germany) and GL-6000-20 scales (CAS, Seoul, Korea), respectively, to the nearest decimal point. Waist circumference (WC) in cm was measured at midpoint between the bottom of rib cage and the top of the iliac crest using non-elastic tape. WHtR was calculated as the WC in cm divided by the height in cm. Obesity was defined by both BMI and WHtR. We used BMI of both ≥25 and ≥30 kg/m^2^ to define obesity based on the original [18] and updated [19] Asia-Pacific standards by the WHO. Defining obesity by using BMI of both ≥25 and ≥30 kg/m^2^ is more useful to compare our study with others in different populations. Based on earlier studies [20, 21], we used the WHtR cut-off point of ≥0.50 to define obesity in both men and women.

### Statistical analysis

We conducted statistical analyses using STATA (version 10.0). All descriptive statistics are presented as frequencies and percentages for categorical variables, and as means and standard deviations for continuous variables. The difference in physical activity levels and prevalence of obesity between genders among different age groups was tested using a Chi-squared test. We used multivariable logistic regression to estimate odds ratios (ORs) and 95% confidence intervals (CIs) of obesity by physical activity levels. All models were adjusted for age group (19–39, 40–64, ≥65 years), academic achievement (elementary school, middle school, high school, college), household income (quartile), smoking status (never, former-smoker, current smoker), alcohol consumption (non-drinker, ≤1 drink/month, 2–4 drinks/month, ≥5 drinks/month), history of diabetes mellitus, dyslipidemia, hypertension, and arthritis, daily energy intake per weight (kg) accounting for gender (quartile), marriage status (not married, married, divorced), and engagement of resistance exercise (≤1 times/week, ≥2 times/week). In addition, menopausal status (yes or no), breast feeding (yes or no), and hysterectomy (yes or no) were additional adjusted in women. Statistical significance was accepted as p<0.05.

## Results

The demographic and obesity measures by age group and gender are shown in Table 1.

**Table 1:** Population characteristics by age group and gender.

	Men					Women					
	All(n = 3350)	19-39(n = 1352)	40-64(n = 1454)	≥65(n = 544)	p^a^	All(n = 3871)	19-39(n = 1537)	40-64(n = 1599)	≥65(n = 735)	p^a^	p^b^
Age (yr)	45.7±15.8	30.7±5.8	50.6±7.1	71.6±5.2	<0.001	46.9±16.5	30.9±5.6	50.5±7.1	72.6±5.6	<0.001	0.014
Weight (kg)	66.8±10.4	69.3±10.9	66.8±9.5	60.8±8.7	<0.001	55.5±8.9	54.8±9.2	57.1±8.5	53.2±8.5	<0.001	<0.001
Height (cm)	169.8±6.6	173.2±5.7	168.7±5.9	164.2±5.6	<0.001	156.4±6.7	160.0±5.4	156.0±5.4	149.9±6.2	<0.001	<0.001
BMI (kg/m^2^)	23.2±3.0	23.1±3.3	23.4±2.9	22.5±2.7	<0.001	22.7±3.4	21.4±3.4	23.5±3.2	23.6±3.2	<0.001	<0.001
WHtR	0.485±0.054	0.465±0.052	0.496±0.050	0.504±0.051	<0.001	0.494±0.069	0.455±0.057	0.508±0.061	0.546±0.062	<0.001	<0.001

Values are expressed as mean ± SD.

BMI: body mass index; WHtR: waist-to-height ratio.

^a^p-value for the differences between age groups in each gender using the analysis of variance (ANOVA).

^b^p-value for the differences between gender using the two-sample t-test.

In both men and women, WHtR increased with increasing age. We also found a similar trend in BMI, except in older men aged ≥65 years with decreased BMI. Between men and women, we found that WHtR is higher in women (0.494) than men (0.485), but BMI was higher in men (23.2 kg/m^2^) than women (22.7 kg/m^2^). However, we found different results in other populations. In a large population study of 36,642 Taiwanese adults aged ≥18 years old, we found that WHtR was higher in men (0.49) than women (0.46), and BMI was also higher in men (24.8 kg/m^2^) than women (22.5 kg/m^2^) [22]. In the British National Survey data, we also found different results compared to our study, indicating that WHtR was higher in men (0.55) than women (0.51), and BMI was also higher in men (27.2 kg/m^2^) than women (26.5 kg/m^2^) in 1776 individuals aged between 19 and 64 years old [23]. In addition, this British study suggests that both WHtR and BMI values are higher in western population than Asian population. Therefore, there are differences in absolute values and patterns of WHtR and BMI between men and women in different populations.

In the joint classification on obesity prevalence by BMI and WHtR in Table 2, we found that 25.6% (n = 1410) of those who are classified ‘non-obese’ by BMI <25 kg/m^2^ (n = 5512) are actually centrally obese by WHtR ≥0.50. This discrepancy was greater in women (28.8%) than men (21.8%). This is an important finding since other studies indicated that normal weight individuals (BMI<25 kg/m^2^) with higher WHtR (≥0.50) had significantly higher cardiometabolic risk factors compared to normal weight individuals with lower WHtR (<0.50) [24, 25]. When we defined obesity by BMI ≥30 kg/m^2^ and WHtR ≥0.50, although specificity (probability that those who are classified ‘non-obese’ by BMI are also centrally non-obese by WHtR) was high, sensitivity (probability that those who are classified ‘obese’ by BMI are actually centrally obese by WHtR) was very low (6.4%), possibly due to a very low prevalence of obesity (2.6%) by BMI ≥30 kg/m^2^ in this population. This result suggests that using BMI ≥30 kg/m^2^ to define obesity in this population may be too strict, which was also reported in a systematic review study [11].

**Table 2:** Joint classification on obesity prevalence by waist-to-height ratio (WHtR) and body mass index (BMI) in men and women.

	All (n = 7221)		Men (n = 3350)		Women (n = 3871)	
	WHtR <0.50	WHtR ≥0.50	WHtR <0.50	WHtR ≥0.50	WHtR <0.50	WHtR ≥0.50
BMI						
<25 kg/m^2^	56.8(4102)	19.5(1410)	59.0(1977)	16.4(550)	54.9(2125)	22.2(860)
≥25 kg/m^2^	2.1(147)	21.6(1562)	2.9(95)	21.7(728)	1.4(52)	21.5(834)
BMI						
<30 kg/m^2^	58.8(4249)	0(0)	61.9(2072)	0(0)	56.3(2177)	0(0)
≥30 kg/m^2^	38.6(2783)	2.6(189)	35.9(1203)	2.2(75)	40.8(1580)	2.9(114)

Values are expressed as % (n).

The prevalence of obesity and meeting the physical activity guidelines by age group and gender are presented in Table 3. We found a higher prevalence of obesity defined by WHtR than BMI in both men and women. Overall, 38.2% men and 43.8% women were obese defined by WHtR, 24.6% men and 22.9% women were obese defined by BMI ≥25 kg/m^2^, and 2.2% men and 2.9% women were obese defined by BMI ≥30 kg/m^2^. We found a higher obesity prevalence in women (43.8%) than men (38.2%) defined by WHtR (p<0.001), but no significant differences in obesity prevalence were found between men and women when obesity was defined by either BMI ≥25 or >30 kg/m^2^. The prevalence of obesity defined by both WHtR ≥0.50 and BMI ≥25 kg/m^2^ consistently increased with increasing age in both men and women, except in older men aged ≥65 years with decreased prevalence of obesity defined by BMI ≥25 kg/m^2^. However, the prevalence of obesity defined by BMI ≥30 kg/m^2^ decreased in men, but increased in women with increasing age, which is less consistent, partly due to lower obesity prevalence (2.6%) when defining obesity by BMI ≥30 kg/m^2^. The percentage of participants who were classified as moderate-to-vigorously active were 75.7% in men and 65.1% in women, respectively. There was a gender difference in the prevalence of meeting the physical activity guidelines (P<0.001), indicating that men are more active than women in this Korean population.

**Table 3:** Prevalence of obesity and meeting the physical activity guidelines by age group and gender.

	Men					Women					
	All(n = 3350)	19-39(n = 1352)	40-64(n = 1454)	≥65(n = 544)	p^a^	All(n = 3871)	19-39(n = 1537)	40-64(n = 1599)	≥65(n = 735)	p^a^	p^b^
Obesity by WHtR ≥0.50	38.2 (1278)	23.2 (314)	46.2 (671)	53.9 (293)	<0.001	43.8 (1694)	18.5 (284)	52.2 (834)	78.4 (576)	<0.001	<0.001
Obesity by BMI ≥25 kg/m^2^	24.6 (823)	24.0 (324)	28.4 (413)	15.8 (86)	<0.001	22.9 (886)	12.8 (196)	28.3 (452)	32.4 (238)	<0.001	0.094
Obesity by BMI ≥30 kg/m^2^	2.2 (75)	3.6 (48)	1.7 (24)	0.6 (3)	<0.001	2.9 (114)	2.3 (36)	3.8 (60)	2.5 (18)	0.044	0.061
Meeting Moderate-to-vigorous PA Guidelines	75.7 (2537)	75.8 (1025)	75.5 (1097)	76.3 (415)	0.923	65.1 (2520)	61.5 (945)	68.8 (1100)	64.6 (475)	<0.001	<0.001

Values are expressed as % (n).

BMI: body mass index; PA: physical activity; WHtR: waist-to-height ratio.

^a^p value for the differences between age groups in each gender using the Chi-square test.

^b^p value for the differences between gender using the Chi-square test.

Table 4 shows the associations between moderate-to-vigorous physical activity and the prevalence of obesity according to WHtR and BMI stratified by gender. In general, the ORs for obesity were lower in the active and very active groups compared to the inactive group after adjusting for potential confounding factors. For the analyses of WHtR, the ORs (95% CIs) for obesity were 0.69 (0.53–0.89) in the active men and 0.76 (0.63–0.91) in the very active men, which were significantly lower, compared to the inactive men (p for trend = 0.007). In women, the OR (95% CI) for obesity was 0.84 (0.71–0.998) in the very active women, which was significantly lower, compared to the inactive women. However, the OR (95% CI) in active women (OR = 0.94; 95% CI = 0.75–1.18) was not significant.

For the analyses of BMI, the only statistically significant results were found in very active men with OR (95% CI) of 0.82 (0.67–0.99) when obesity was defined by BMI ≥25 kg/m^2^ and active men with OR (95% CI) of 0.40 (0.15–0.98) when obesity was defined by BMI ≥30 kg/m^2^. However, we found no statistically significant result in women when obesity was defined by BMI ≥25 or ≥30 kg/m^2^. These results in Table 4 indicate more consistent findings on the associations between physical activity and obesity when obesity was defined by WHtR rather than BMI in both men and women.

**Table 4:** Associations between moderate-to-vigorous physical activity and obesity by waist-to-height ratio (WHtR) and body mass index (BMI) in men and women.

	Men	Women
	OR (95% CI)^a^	OR (95% CI)^a^
Obesity by WHtR ≥0.50		
Moderate-to-vigorous PA		
Inactive	1.00	1.00
Active	0.69 (0.53–0.89)	0.94 (0.75–1.18)
Very active	0.76 (0.63–0.91)	0.84 (0.71–0.998)
p for trend	0.007	0.046
Obesity by BMI ≥25 kg/m^2^		
Moderate-to-vigorous PA		
Inactive	1.00	1.00
Active	0.78 (0.59–1.03)	0.92 (0.72–1.18)
Very active	0.82 (0.67–0.99)	0.96 (0.80–1.15)
p for trend	0.060	0.658
Obesity by BMI ≥30 kg/m^2^		
Moderate-to-vigorous PA		
Inactive	1.00	1.00
Active	0.40 (0.15–0.98)	0.96 (0.55–1.68)
Very active	0.90 (0.52–1.56)	0.80 (0.52–1.23)
p for trend	0.978	0.296

CI: confidence interval; OR: odds ratio; PA: physical activity.

^a^Adjusted for age group (19–39, 40–64, ≥65 years), academic achievement (elementary school, middle school, high school, college), household income (quartile), smoking status (never, former-smoker, current smoker), alcohol consumption (non-drinker, ≤1 drink/month, 2–4 drinks/month, ≥5 drinks/month), history of diabetes mellitus, dyslipidemia, hypertension, and arthritis, daily energy intake per weight (kg) accounting for gender (quartile), marriage status (not married, married, divorced), and engagement of resistance exercise (≤1/week, ≥2/week) for both men and women, and menopausal status (yes or no), breast feeding (yes or no), and hysterectomy (yes or no) for women.

## Discussion

We found that the prevalence of obesity was very different between the two obesity measures of WHtR and BMI in Korean adult population. The prevalence of obesity according to WHtR was greater than the prevalence of obesity according to BMI in both men and women. Also, the greatest discrepancy in the prevalence of obesity between WHtR and BMI was found in the older adults aged ≥65 years old (53.9% by WHtR vs. 15.8% and 0.6% by BMI ≥25 and ≥30 kg/m^2^, respectively, in men, and 78.4% by WHtR vs. 32.4% and 2.5% by BMI ≥25 and ≥30 kg/m^2^, respectively, in women). The prevalence of obesity according to WHtR increased more dramatically with increasing age. This dramatic increase in obesity prevalence defined by WHtR in older adults could be explained by the fact that there is an age-related loss of lean body mass [26] and redistribution of adipose tissue to central and intra-abdominal compartments [27].

As mentioned previously, BMI cannot distinguish muscle from fat. There are other measure of abdominal obesity such as WC and waist-hip ratio (WHR). Although these are simple measures of abdominal obesity, they do not take into account differences in height between individuals. Compared to the other indices, WHtR is reported to be more sensitive [9, 28]. Several studies have shown that WHtR is a better predictor of cardiovascular disease risk factors than BMI [29, 30]. Also, WHtR may predict cardiometabolic risk better than other indices such as WC or WHR [20, 31]. Moreover, WHtR is more practical as it has been used as the basis of a consumer-friendly chart [32]. The cut-off value of WHtR (≥0.50) to define obesity has been used to support the simple public health message, “keep your waist circumference to less than half of your height.” [33, 34].

As mentioned above, given the same BMI, Asians have a higher percentage of body fat, abdominal obesity, higher levels of intramyocellular lipids and/or a higher liver fat content compared to Caucasians [14, 15]. These characteristics can contribute to a higher predisposition to insulin resistance and other metabolic diseases at a lesser degree of obesity than Caucasians. Based on these ethnic differences, more sensitive measure for abdominal obesity such as WHtR is required for more accurate prediction of potential cardiometabolic diseases in Asian populations.

Associations between physical activity and obesity are well documented [35]. However, in this study, we found that the inverse relationship of physical activity with obesity by WHtR was more significant than that of physical activity with obesity by BMI, especially in women. We found consistently significant associations between moderate-to-vigorous physical activity and obesity by WHtR in both men and women (both p for trend<0.05). However, when we defined obesity by BMI, we found partially significant associations between physical activity and obesity in men (only in very active men using BMI ≥25 kg/m^2^ and only in active men using BMI ≥30 kg/m^2^ to define obesity), and no significant association was found in women (Table 4). This result suggests that WHtR is possibly a more adequate measure of obesity than BMI, specifically in women. One of the possible explanations of the poor correlation between BMI and physical activity would be that BMI measures muscle as well as fat, and some people with high BMI would be over-muscular and not over-fat. Physically active adults may reduce body fat, preferentially more from the abdominal area [36], so their WHtR would be lower. This supports our result of the inverse association between physical activity and obesity defined by WHtR.

In this study, moderate-to-vigorous physical activity using the recommended cut-off values of the current guidelines was significantly associated with a lower prevalence of obesity, especially in men using both WHtR and BMI to define obesity. However, we found a significant association between moderate-to-vigorous physical activity and obesity defined by WHtR, but not by BMI, in women. The difference in results between genders is not clearly understood. However, it is probably related to the difference in the accumulation of excess fat in the abdominal area between men and women [37]. Also, as mentioned earlier, women are less likely to be over-muscular, which explains the differing results between genders. In women, we found that having twice as much physical activity (very active group) than the recommended minimal amount of activity (active group) was associated with the significantly decreased odds ratio of obesity by WHtR (Table 4), which suggests that more physical activity might be needed to prevent obesity in women [38, 39]. This is supported by the physical activity recommendations of the International Association for the Study of Obesity: to prevent the transition from overweight to obesity, more physical activity (at least 45–60 min/day of moderate activity) is required, and to prevent weight regain in obese individuals, more physical activity (60–90 min/day of moderate activity) is also required [40].

We found somewhat increased ORs for obesity in ‘very active’ men compared to ORs in ‘active’ men in both WHtR and BMI defined obesity. Although it is difficult to find explanations in this cross-sectional analysis, a reverse causation could be possible that more obese individuals are more motivated to be ‘very active’ in this population, especially in men. Another possible explanation would be related to the exercise and food compensation hypothesis that more active people reward themselves for being active by consuming more food after exercise, and this increased energy intake may lead to decreased weight reduction [41]. However, more studies are needed on this important topic.

A major limitation of the study is its cross-sectional design, which could not ascertain a causal relationship between physical activity and obesity. However, we excluded those participants who engaged regular physical activity less than one year to investigate more long term associations between physical activity and obesity. Self-reported physical activity and a lack of criterion measure to determine body fatness (e.g., using DXA) may be other limitations. However, to our knowledge, this is the first national study on the associations between physical activity and obesity in the representative Korean population. We also defined obesity by BMI as well as WHtR, which is more valid and predictive of future cardiometabolic diseases, especially in Asian populations. To investigate and compare causal associations between physical activity and different obesity measures in various populations, intervention studies are clearly warranted.
